# RNA protects a nucleoprotein complex against radiation damage

**DOI:** 10.1107/S2059798316003351

**Published:** 2016-04-26

**Authors:** Charles S. Bury, John E. McGeehan, Alfred A. Antson, Ian Carmichael, Markus Gerstel, Mikhail B. Shevtsov, Elspeth F. Garman

**Affiliations:** a Laboratory of Molecular Biophysics, Department of Biochemistry, University of Oxford, South Parks Road, Oxford OX1 3QU, England; b Molecular Biophysics, Institute of Biomedical and Biomolecular Sciences, University of Portsmouth, King Henry I Street, Portsmouth PO1 2DY, England; c York Structural Biology Laboratory, Department of Chemistry, University of York, York Y010 5DD, England; d Notre Dame Radiation Laboratory, University of Notre Dame, Notre Dame, IN 46556, USA; e Laboratory of Structural Biology of GPCRs, Moscow Institute of Physics and Technology, Dolgoprudniy 141700, Russian Federation

**Keywords:** radiation damage, protein–RNA complex, electron difference density, specific damage, decarboxylation

## Abstract

Radiation damage during macromolecular X-ray crystallographic data collection is still the main impediment for many macromolecular structure determinations. Even when an eventual model results from the crystallographic pipeline, the manifestations of radiation-induced structural and conformation changes, the so-called specific damage, within crystalline macromolecules can lead to false interpretations of biological mechanisms. Although this has been well characterized within protein crystals, far less is known about specific damage effects within the larger class of nucleoprotein complexes. Here, a methodology has been developed whereby per-atom density changes could be quantified with increasing dose over a wide (1.3–25.0 MGy) range and at higher resolution (1.98 Å) than the previous systematic specific damage study on a protein–DNA complex. Specific damage manifestations were determined within the large *trp* RNA-binding attenuation protein (TRAP) bound to a single-stranded RNA that forms a belt around the protein. Over a large dose range, the RNA was found to be far less susceptible to radiation-induced chemical changes than the protein. The availability of two TRAP molecules in the asymmetric unit, of which only one contained bound RNA, allowed a controlled investigation into the exact role of RNA binding in protein specific damage susceptibility. The 11-fold symmetry within each TRAP ring permitted statistically significant analysis of the Glu and Asp damage patterns, with RNA binding unexpectedly being observed to protect these otherwise highly sensitive residues within the 11 RNA-binding pockets distributed around the outside of the protein molecule. Additionally, the method enabled a quantification of the reduction in radiation-induced Lys and Phe disordering upon RNA binding directly from the electron density.

## Abstract 2

Systematic analysis of radiation damage within a protein–RNA complex over a large dose range (1.3–25 MGy) reveals significant differential susceptibility of RNA and protein. A new method of difference electron-density quantification is presented.

## 1. Introduction   

With the wide use of high-flux third-generation synchrotron sources, radiation damage (RD) has once again become a dominant reason for the failure of structure determination using macromolecular crystallography (MX) in experiments conducted both at room temperature and under cryocooled conditions (100 K). Significant progress has been made in recent years in understanding the inevitable manifestations of X-ray-induced RD within protein crystals, and there is now a body of literature on possible strategies to mitigate the effects of RD (*e.g.* Zeldin, Brockhauser *et al.*, 2013 ▸; Bourenkov & Popov, 2010 ▸). However, there is still no general consensus within the field on how to minimize RD during MX data collection, and debates on the dependence of RD progression on incident X-ray energy (Shimizu *et al.*, 2007 ▸; Liebschner *et al.*, 2015 ▸) and the efficacy of radical scavengers (Allan *et al.*, 2013 ▸) have yet to be resolved.

RD manifests in two forms. *Global radiation damage* is observed within reciprocal space as the overall decay of the summed intensity of reflections detected within the diffraction pattern as dose increases (Garman, 2010 ▸; Murray & Garman, 2002 ▸). Dose is defined as the absorbed energy per unit mass of crystal in grays (Gy; 1 Gy = 1 J kg^−1^), and is the metric against which damage progression should be monitored during MX data collection, as opposed to time. At 100 K, an experimental dose limit of 30 MGy has been recommended as an upper limit beyond which the biological information derived from any macromolecular crystal may be compromised (Owen *et al.*, 2006 ▸).

*Specific radiation damage* (SRD) is observed in the real-space electron density, and has been detected at much lower doses than any observable decay in the intensity of reflections. Indeed, the C—Se bond in selenomethionine, the stability of which is key for the success of experimental phasing methods, can be cleaved at a dose as low as 2 MGy for a crystal maintained at 100 K (Holton, 2007 ▸). SRD has been well characterized in a large range of proteins, and is seen to follow a reproducible order: metallo-centre reduction, disulfide-bond cleavage, acidic residue decarboxylation and methionine methylthio cleavage (Ravelli & McSweeney, 2000 ▸; Burmeister, 2000 ▸; Weik *et al.*, 2000 ▸; Yano *et al.*, 2005 ▸). Furthermore, damage susceptibility within each residue type follows a preferential ordering influenced by a combination of local environment factors (solvent accessibility, conformational strain, proximity to active sites/high X-ray cross-section atoms; Holton, 2009 ▸). Deconvoluting the individual roles of these parameters has been surprisingly challenging, with factors such as solvent accessibility currently under active investigation (Weik *et al.*, 2000 ▸; Fioravanti *et al.*, 2007 ▸; Gerstel *et al.*, 2015 ▸).

There are a number of cases where SRD manifestations have compromised the biological information extracted from MX-determined structures at much lower doses than the recommended 30 MGy limit, leading to false structural interpretations of protein mechanisms. Active-site residues appear to be particularly susceptible, particularly for photosensitive proteins and in instances where chemical strain is an intrinsic feature of the reaction mechanism. For instance, structure determination of the purple membrane protein bacterio­rhodopsin required careful corrections for radiation-induced structural changes before the correct photosensitive intermediate states could be isolated (Matsui *et al.*, 2002 ▸). The significant chemical strain required for catalysis within the active site of phosphoserine aminotransferase has been observed to diminish during X-ray exposure (Dubnovitsky *et al.*, 2005 ▸).

Since the majority of SRD studies to date have focused on proteins, much less is known about the effects of X-ray irradiation on the wider class of crystalline nucleoprotein complexes or how to correct for such radiation-induced structural changes. Understanding RD to such complexes is crucial, since DNA is rarely naked within a cell, instead dynamically interacting with proteins, facilitating replication, transcription, modification and DNA repair. As of early 2016, >5400 nucleoprotein complex structures have been deposited within the PDB, with 91% solved by MX. It is essential to understand how these increasingly complex macromolecular structures are affected by the radiation used to solve them. Nucleoproteins also represent one of the main targets of radiotherapy, and an insight into the damage mechanisms induced by X-ray irradiation could inform innovative treatments.

When a typical macromolecular crystal is irradiated with ionizing X-rays, each photoelectron produced *via* interactions with both the macromolecule (direct damage) and solvent (indirect damage) can induce cascades of up to 500 secondary low-energy electrons (LEEs) that are capable of inducing further ionizations. Investigations on sub-ionization-level LEEs (0–15 eV) interacting with both dried and aqueous oligonucleotides (Alizadeh & Sanche, 2014 ▸; Simons, 2006 ▸) concluded that resonant electron attachment to DNA bases and the sugar-phosphate backbone could lead to the preferential cleavage of strong (∼4 eV, 385 kJ mol^−1^) sugar-phosphate C—O covalent bonds within the DNA backbone and then base-sugar N_1_—C bonds, eventually leading to single-strand breakages (SSBs; Ptasińska & Sanche, 2007 ▸). Electrons have been shown to be mobile at 77 K by electron spin resonance spectroscopy studies (Symons, 1997 ▸; Jones *et al.*, 1987 ▸), with rapid electron quantum tunnelling and positive hole migration along the protein backbone and through stacked DNA bases indicated as a dominant mechanism by which oxidative and reductive damage localizes at distances from initial ionization sites at 100 K (O’Neill *et al.*, 2002 ▸).

The investigation of naturally forming nucleoprotein complexes circumvents the inherent challenges in making controlled comparisons of damage mechanisms between protein and nucleic acids crystallized separately. Recently, for a well characterized bacterial protein–DNA complex (C.Esp1396I; PDB entry 3clc; resolution 2.8 Å; McGeehan *et al.*, 2008 ▸) it was concluded that over a wide dose range (2.1–44.6 MGy) the protein was far more susceptible to SRD than the DNA within the crystal (Bury *et al.*, 2015 ▸). Only at doses above 20 MGy were precursors of phosphodiester-bond cleavage observed within AT-rich regions of the 35-mer DNA.

**Figure 1:**
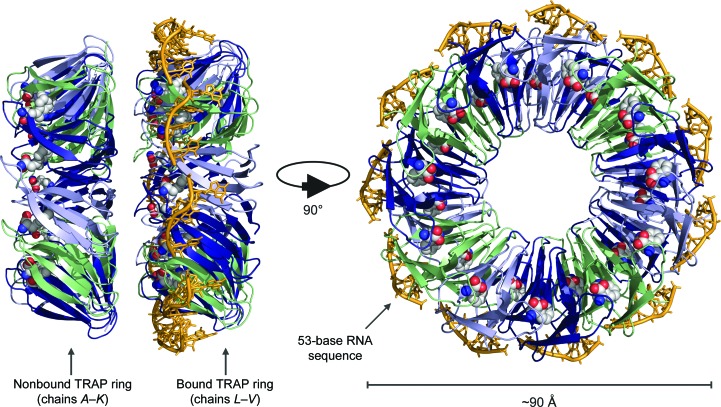
The TRAP–(GAGUU)_10_GAG complex asymmetric unit (PDB entry 1gtf; Hopcroft *et al.*, 2002 ▸). Bound tryptophan ligands are represented as coloured spheres. RNA is shown is yellow.

For crystalline complexes such as C.Esp1396I, whether the protein is intrinsically more susceptible to X-ray-induced damage or whether the protein scavenges electrons to protect the DNA remains unclear in the absence of a non-nucleic acid-bound protein control obtained under exactly the same crystallization and data-collection conditions. To monitor the effects of nucleic acid binding on protein damage susceptibility, a crystal containing two protein molecules per asymmetric unit, only one of which was bound to RNA, is reported here (Fig. 1 ▸). Using newly developed methodology, we present a controlled SRD investigation at 1.98 Å resolution using a large (∼91 kDa) crystalline protein–RNA complex: *trp* RNA-binding attenuation protein (TRAP) bound to a 53 bp RNA sequence (GAGUU)_10_GAG (PDB entry 1gtf; Hopcroft *et al.*, 2002 ▸). TRAP consists of 11 identical subunits assembled into a ring with 11-fold rotational symmetry. It binds with high affinity (*K*_d_ ≃ 1.0 n*M*) to RNA segments containing 11 GAG/UAG triplets separated by two or three spacer nucleotides (Elliott *et al.*, 2001 ▸) to regulate the transcription of tryptophan biosynthetic genes in *Bacillus subtilis* (Antson *et al.*, 1999 ▸). In this structure, the bases of the G1-A2-G3 nucleotides form direct hydrogen bonds to the protein, unlike the U4-U5 nucleotides, which appear to be more flexible.

Ten successive 1.98 Å resolution MX data sets were collected from the same TRAP–RNA crystal to analyse X-ray-induced structural changes over a large dose range (*d*_1_ = 1.3 MGy to *d*_10_ = 25.0 MGy). To avoid the previous necessity for visual inspection of electron-density maps to detect SRD sites, a computational approach was designed to quantify the electron-density change for each refined atom with increasing dose, thus providing a rapid systematic method for SRD study on such large multimeric complexes. By employing the high 11-fold structural symmetry within each TRAP macromolecule, this approach permitted a thorough statistical quantification of the RD effects of RNA binding to TRAP.

## 2. Materials and methods   

### 2.1. RNA synthesis and protein preparation   

As previously described (Hopcroft *et al.*, 2002 ▸), the 53-base RNA (GAGUU)_10_GAG was synthesized by *in vitro* transcription with T7 RNA polymerase and gel-purified. TRAP from *B. stearothermophilus* was overexpressed in *Escherichia coli* and purified.

### 2.2. Crystallization   

TRAP–RNA crystals were prepared using a previously established hanging-drop crystallization protocol (Antson *et al.*, 1999 ▸). By using a 2:1 molar ratio of TRAP to RNA, crystals successfully formed from the protein–RNA complex (∼15 mg ml^−1^) in a solution containing 70 m*M* potassium phosphate pH 7.8 and 10 m*M* l-tryptophan. The reservoir consisted of 0.2 *M* potassium glutamate, 50 m*M* triethanol­amine pH 8.0, 10 m*M* MgCl_2_, 8–11% monomethyl ether PEG 2000. In order to accelerate crystallization, a further gradient was induced by adding 0.4 *M* KCl to the reservoir after 1.5 µl protein solution had been mixed with an equal volume of the reservoir solution. Wedge-shaped crystals of approximate length 70 µm (longest dimension) grew within 3 d and were vitrified and stored in liquid nitrogen immediately after growth. The cryosolution consisted of 12% monomethyl ether PEG 2000, 30 m*M* triethanolamine pH 8.0, 6 m*M* l-tryptophan, 0.1 *M* potassium glutamate, 35 m*M* potassium phosphate pH 7.8, 5 m*M* MgCl_2_ with 25% 2-methyl-2,4-pentanediol (MPD) included as a cryoprotectant.

### 2.3. X-ray data collection   

Data were collected at 100 K from a wedge-shaped TRAP–RNA crystal of approximate dimensions 70 × 20 × 40 µm (see Supplementary Fig. S2) on beamline ID14-4 at the ESRF using an incident wavelength of 0.940 Å (13.2 keV) and an ADSC Q315R mosaic CCD detector at 304.5 mm from the crystal throughout the data collection. The beam size was slitted to 0.100 mm (vertical) × 0.160 mm (horizontal), with a uniformly distributed profile, such that the crystal was completely bathed within the beam throughout data collection. Ten successive (1.98 Å resolution) 180° data sets (with Δφ = 1°) were collected over the same angular range from a TRAP–RNA crystal at 28.9% beam transmission. The TRAP–RNA macromolecule crystallized in space group *C*2, with unit-cell parameters *a* = 140.9, *b* = 110.9, *c* = 137.8 Å, α = γ = 90, β = 137.8° (the values quoted are for the first data set; see Supplementary Table S1 for subsequent values). For the first nine data sets the attenuated flux was recorded to be ∼5 × 10^11^ photons s^−1^. A beam refill took place immediately before data set 10, requiring a flux-scale factor increase of 1.42 to be applied, based on the ratio of observed relative intensity *I*_D_/*I*_1_ at data set 10 to that extrapolated from data set 9.

### 2.4. Dose calculation   

*RADDOSE*-3*D* (Zeldin, Gerstel *et al.*, 2013 ▸) was used to calculate the absorbed dose distribution during each data set (see input file; Supplementary Figs. S1 and S2). The crystal composition was calculated from the deposited TRAP–RNA structure (PDB entry 1gtf; Hopcroft *et al.*, 2002 ▸). Crystal absorption coefficients were calculated in *RADDOSE*-3*D* using the concentration (mmol l^−1^) of solvent heavy elements from the crystallization conditions. The beam-intensity profile was modelled as a uniform (‘top-hat’) distribution. The diffraction-weighted dose (DWD) values (Zeldin, Brock­hauser *et al.*, 2013 ▸) are given in Supplementary Table S1.

### 2.5. Data processing and model refinement   

Each data set was integrated using *iMosflm* (Leslie & Powell, 2007 ▸) and was scaled using *AIMLESS* (Evans & Murshudov, 2013 ▸; Winn *et al.*, 2011 ▸) using the same 5% *R*_free_ set of test reflections for each data set. To phase the structure obtained from the first data set, molecular replacement was carried out with *Phaser* (McCoy *et al.*, 2007 ▸), using an identical TRAP–RNA structure (PDB entry 1gtf; resolution 1.75 Å; Hopcroft *et al.*, 2002 ▸) as a search model. The resulting TRAP–RNA structure (TR1) was refined using *REFMAC*5 (Murshudov *et al.*, 2011 ▸), initially using rigid-body refinement, followed by repeated cycles of restrained, TLS and isotropic *B*-factor refinement, coupled with visual inspection in *Coot* (Emsley *et al.*, 2010 ▸). TR1 was refined to 1.98 Å resolution, with a dimeric assembly of non-RNA-bound and RNA-bound TRAP rings within the asymmetric unit. Consistent with previous structures of the TRAP–RNA complex, the RNA sequence termini were not observed within the 2*F*_o_ − *F*_c_ map; the first spacer (U4) was then modelled at all 11 repeats around the TRAP ring and the second spacer (U5) was omitted from the final refined structure. For the later data sets, the observed structure-factor amplitudes from each separately scaled data set (output from *AIMLESS*) were combined with the phases of TR1 and the resulting higher-dose model was refined with *phenix.refine* (Adams *et al.*, 2010 ▸) using only rigid-body and isotropic *B*-factor refinement. During this refinement, the TRAP–RNA complex and nonbound TRAP ring were treated as two separate rigid bodies within the asymmetric unit. Supplementary Table S1 shows the relevant summary statistics.

### 2.6. *D*_loss_ metric calculation   

The *CCP*4 program *CAD* was used to create a series of nine merged .mtz files combining observed structure-factor amplitudes for the first data set *F*_obs_(*d*_1_) with each later data set *F*_obs_(*d*_*n*_) (for *n* = 2, …, 10). All later data sets were scaled against the initial low-dose data set in *SCALEIT*. For each data set an atom-tagged .map file was generated using the ATMMAP mode in *SFALL* (Winn *et al.*, 2011 ▸). A full set of nine Fourier difference maps *F*_obs_(*d*_*n*_) − *F*_obs_(*d*_1_) were calculated using *FFT* (Ten Eyck, 1973 ▸) over the full TRAP–RNA unit-cell dimensions, with the same grid-sampling dimensions as the atom-tagged .map file. All maps were cropped to the TRAP asymmetric unit in *MAPMASK*. Comparing the atom-tagged .map file and *F*_obs_(*d*_*n*_) − *F*_obs_(*d*_1_) difference map at each dose, each refined atom was assigned a set of density-change values *X*. The maximum density-loss metric, *D*_loss_ (units of e Å^−3^), was calculated to quantify the per-atom electron-density decay at each dose, assigned as the absolute magnitude of the most negative Fourier difference map voxel value in a local volume around each atom as defined by the set X.

### 2.7. Model system calculation   

Model calculations were run for the simple amino acids glutamate and aspartate. In order to avoid decarboxylation at the C-terminus instead of the side chain on the C^α^ atom, the C-terminus of each amino acid was methylated. While the structures of the closed shell acids are well known, the same is not true of those in the oxidized state. The quantum-chemical calculations employed were chosen to provide a satisfactory description of the structure of such radical species and also provide a reliable estimation of the relative C—C(O_2_) bond strengths, which are otherwise not available.

Structures of methyl-terminated (at the N- and C-termini) carboxylates were determined using analytic energy gradients with density functional theory (B3LYP functional; Becke, 1993 ▸) and a flexible basis set of polarized valence triple-zeta size with diffuse functions on the non-H atoms [6-311+G(d,p)] in the *Gaussian* 09 computational chemistry package (Frisch *et al.*, 2009 ▸). The stationary points obtained were characterized as at least local minima by examination of the associated analytic Hessian. Effects of the medium were modelled using a dielectric cavity approach (Tomasi *et al.*, 1999 ▸) parameterized for water.

## 3. Results   

### 3.1. Per-atom quantification of electron density   

To quantify the exact effects of nucleic acid binding to a protein on SRD susceptibility, a high-throughput and automated pipeline was created to systematically calculate the electron-density change for every refined atom within the TRAP–RNA structure as a function of dose. This provides an atom-specific quantification of density–dose dynamics, which was previously lacking within the field. Previous studies have characterized SRD sites by reporting magnitudes of *F*_obs_(*d*_*n*_) − *F*_obs_(*d*_1_) Fourier difference map peaks in terms of the sigma (σ) contour level (the number of standard deviations from the mean map electron-density value) at which peaks become visible. However, these σ levels depend on the standard deviation values of the map, which can deviate between data sets, and are thus unsuitable for quantitative comparison of density between different dose data sets. Instead, we use here a maximum density-loss metric (*D*_loss_), which is the per-atom equivalent of the magnitude of these negative Fourier difference map peaks in units of e Å^−3^. Large positive *D*_loss_ values indicate radiation-induced atomic disordering reproducibly throughout the unit cells with respect to the initial low-dose data set.

**Figure 2:**
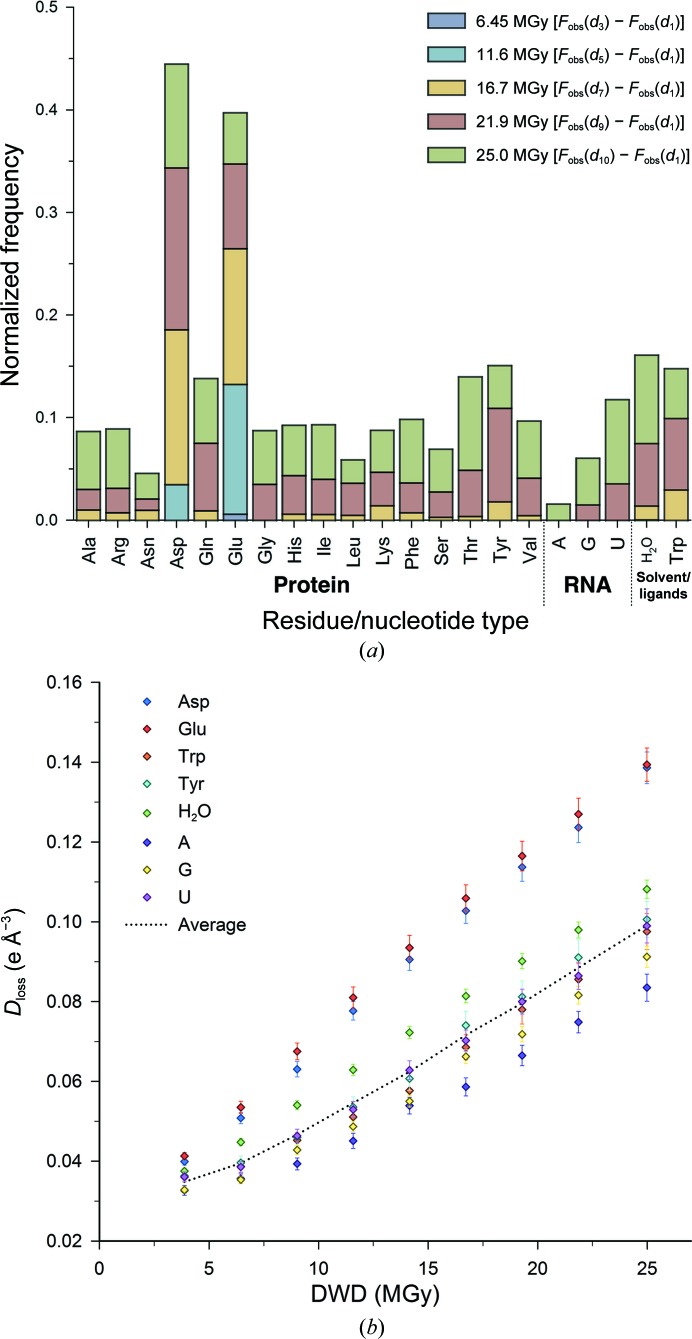
(*a*) Electron-density loss sites as indicated by *D*_loss_ in the TRAP–RNA complex crystal by residue/nucleotide type for five doses [sites determined above the 4× average *D*_loss_ threshold, calculated over the TRAP–RNA structure for the first difference map: *F*_obs_(*d*_2_) − *F*_obs_(*d*_1_)]. Cumulative frequencies are normalized to both the total number of non-H atoms per residue/nucleotide and the total number of that residue/nucleotide type present. (*b*) Average *D*_loss_ for each residue/nucleotide type with respect to the DWD (diffraction-weighted dose; Zeldin, Brock­hauser *et al.*, 2013 ▸). 95% confidence intervals (CI) are shown. Only a subset of key TRAP residue types are included. The average *D*_loss_ (calculated over the whole TRAP asymmetric unit) is shown at each dose (dashed line).

**Figure 3:**
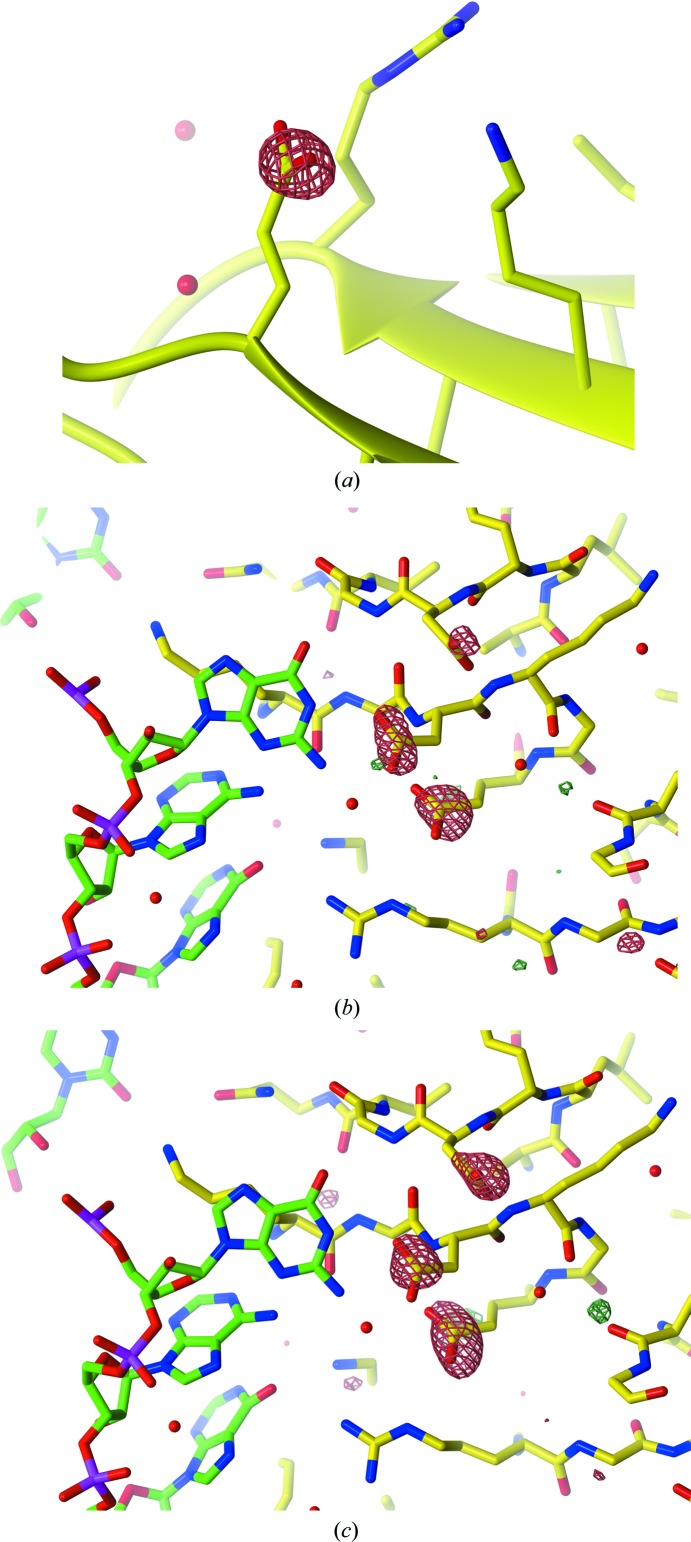
*F*_obs_(*d*_*n*_) − *F*_obs_(*d*_1_) Fourier difference maps for (*a*) *n* = 2 (3.9 MGy), (*b*) *n* = 3 (6.5 MGy) and (*c*) *n* = 7 (16.7 MGy) contoured at ±4σ (*a*) and ±3.5σ (*b*, *c*). In (*a*) clear difference density is observed around the Glu42 carboxyl side chain in chain *H*, within the lowest dose difference map at *d*_2_ = 3.9 MGy. Radiation-induced protein disordering is evident across the large dose range (*b*, *c*); in comparison, no clear deterioration of the RNA density was observed.

For each TRAP–RNA data set, the *D*_loss_ metric successfully identified the recognized forms of protein SRD (Fig. 2 ▸ *a*), with clear Glu and Asp side-chain decarboxylation even in the first difference map calculated (3.9 MGy; Fig. 3 ▸ *a*). The main sequence of TRAP does not contain any Trp and Cys residues (and thus contains no disulfide bonds). The substrate Trp amino-acid ligands also exhibited disordering of the free terminal carboxyl groups at higher doses (Fig. 2 ▸ *a*); however, no clear Fourier difference peaks could be observed visually. Even for radiation-insensitive residues (*e.g.* Gly) the average *D*_loss_ increases with dose: this is the effect of global radiation damage, since as dose increases the electron density associated with each refined atom becomes weaker as the atomic occupancy decreases (Fig. 2 ▸ *b*). Only Glu and Asp residues exhibit a rate of *D*_loss_ increase that consistently exceeds the average decay (Fig. 2 ▸ *b*, dashed line) at each dose. Additionally, the density surrounding ordered solvent molecules was determined to significantly diminish with increasing dose (Fig. 2 ▸ *b*). The rate of *D*_loss_ (attributed to side-chain decarboxylation) was consistently larger for Glu compared with Asp residues over the large dose range (Fig. 2 ▸ *b* and Supplementary Fig. S3); this observation is consistent with our calculations on model systems (see above) that suggest that, without considering differential hydrogen-bonding environments, CO_2_ loss is more exothermic by around 8 kJ mol^−1^ from oxidized Glu residues than from their Asp counterparts.

### 3.2. RNA is less susceptible to electron-density loss than protein within the TRAP–RNA complex   

Visual inspection of Fourier difference maps illustrated the clear lack of RNA electron-density degradation with increasing dose compared with the obvious protein damage manifestations (Figs. 3 ▸ *b* and 3 ▸ *c*). Only at the highest doses investigated (>20 MGy) was density loss observed at the RNA phosphate and C—O bonds of the phosphodiester backbone. However, the median *D*_loss_ was lower by a factor of >2 for RNA P atoms than for Glu and Asp side-chain groups at 25.0 MGy (Supplementary Fig. S4), and furthermore could not be numerically distinguished from Gly C^α^ atoms within TRAP, which are not radiation-sensitive at the doses tested here (Supplementary Fig. S3).

### 3.3. RNA binding protects radiation-sensitive residues   

**Figure 4:**
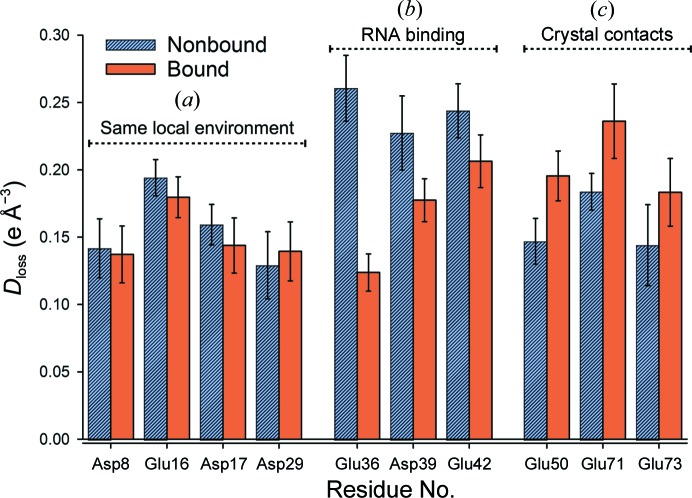
*D*_loss_ calculated for all side-chain carboxyl group Glu C^δ^ and Asp C^γ^ atoms within the TRAP–RNA complex for a dose of 19.3 MGy (*d*_8_). Residues have been grouped by amino-acid number, and split into bound and nonbound groupings, with each bar representing the mean calculated over 11 equivalent atoms around a TRAP ring. Whiskers indicate 95% CI. The *D*_loss_ behaviour shown here was consistently exhibited across the entire investigated dose range.

For the large number of acidic residues per TRAP ring (four Asp and six Glu residues per protein monomer), a strong dependence of decarboxylation susceptibility on local environment was observed (Fig. 4 ▸). For each Glu C^δ^ or Asp C^γ^ atom, *D*_loss_ provided a direct measure of the rate of side-chain carboxyl-group disordering and subsequent decarboxylation. For acidic residues with no differing interactions between nonbound and bound TRAP (Fig. 4 ▸ *a*), similar damage was apparent between the two rings within the asymmetric unit, as expected. However, TRAP residues directly on the RNA-binding interfaces exhibited greater damage accumulation in nonbound TRAP (Fig. 4 ▸ *b*), and for residues at the ring–ring interfaces (where crystal contacts were detected) bound TRAP exhibited enhanced SRD accumulation (Fig. 4 ▸ *c*).

**Figure 5:**
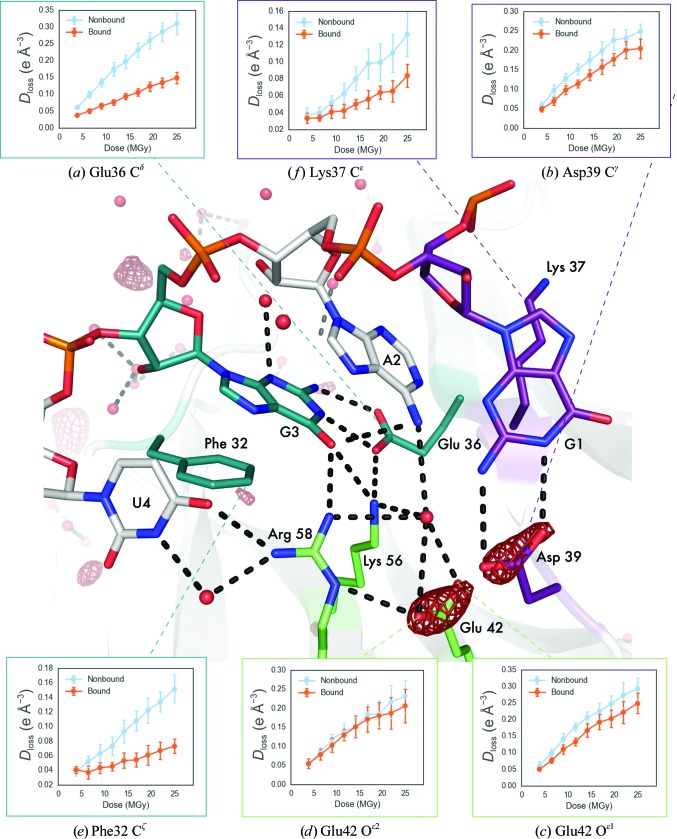
*D*_loss_ against dose for (*a*) Glu36 C^δ^, (*b*) Asp39 C^γ^, (*c*) Glu42 O^∊1^, (*d*) Glu42 O^∊2^, (*e*) Phe32 C^ζ^ and (*f*) Lys37 C^∊^ atoms. 95% CI are included for each set of 11 equivalent atoms grouped as bound/nonbound. RNA-binding interface interactions are shown for TRAP chain *N*, with the *F*_obs_(*d*_7_) − *F*_obs_(*d*_1_) Fourier difference map (dose 16.7 MGy) overlaid and contoured at a ±4σ level.

Three acidic residues (Glu36, Asp39 and Glu42) are involved in RNA interactions within each of the 11 TRAP ring subunits, and Fig. 5 ▸ shows their density changes with increasing dose. Hotelling’s T-squared test (the multivariate counterpart of Student’s t-test) was used to reject the null hypothesis that the means of the *D*_loss_ metric were equal for the bound and nonbound groups in Fig. 5 ▸.

A significant reduction in *D*_loss_ is seen for Glu36 in RNA-bound compared with nonbound TRAP, indicative of a lower rate of side-chain decarboxylation (Fig. 5 ▸ *a*; *p* = 6.06 × 10^−5^). For each TRAP ring subunit, the Glu36 side-chain carboxyl group accepts a pair of hydrogen bonds from the two N atoms of the G3 RNA base. In our analysis, Asp39 in the TRAP–(GAGUU)_10_GAG structure appears to exhibit two distinct hydrogen bonds to the G1 base within each of the 11 TRAP–RNA interfaces, as does Glu36 to G3; however, the reduction in density disordering upon RNA binding is far less significant for Asp39 than for Glu36 (Fig. 5 ▸ *b*, *p* = 0.0925).

### 3.4. RNA binding reduces radiation-induced disorder on the atomic scale   

One oxygen (O^∊1^) of Glu42 appears to form a hydrogen bond to a nearby water within each TRAP RNA-binding pocket, with the other (O^∊2^) being involved in a salt-bridge interaction with Arg58 (Hopcroft *et al.*, 2002 ▸; Antson *et al.*, 1999 ▸). Salt-bridge interactions have previously been suggested to reduce the glutamate decarboxylation rate within the large (∼62.4 kDa) myrosinase protein structure (Burmeister, 2000 ▸). A significant difference was observed between the *D*_loss_ dynamics for the nonbound/bound Glu42 O^∊1^ atoms (Fig. 5 ▸ *c*; *p* = 0.007) but not for the Glu42 O^∊2^ atoms (Fig. 5 ▸ *d*; *p* = 0.239), indicating that the stabilizing strength of this salt-bridge interaction was conserved upon RNA binding and that the water-mediated hydrogen bond had a greater relative susceptibility to atomic disordering in the absence of RNA. The density-change dynamics were statistically indistinguishable between bound and nonbound TRAP for each Glu42 carboxyl group C^δ^ atom (*p* = 0.435), indicating that upon RNA binding the conserved salt-bridge interaction ultimately dictated the overall Glu42 decarboxylation rate.

The RNA-stabilizing effect was not restricted to radiation-sensitive acidic residues. The side chain of Phe32 stacks against the G3 base within the 11 TRAP RNA-binding interfaces (Antson *et al.*, 1999 ▸). With increasing dose, the *D*_loss_ associated with the Phe32 side chain was significantly reduced upon RNA binding (Fig. 5 ▸ *e*; Phe32 C^ζ^; *p* = 0.0014), an indication that radiation-induced conformation disordering of Phe32 had been reduced. The extended aliphatic Lys37 side chain stacks against the nearby G1 base, making a series of nonpolar contacts within each RNA-binding interface. The *D*_loss_ for Lys37 side-chain atoms was also reduced when stacked against the G1 base (Fig. 5 ▸ *f*; *p* = 0.0243 for Lys37 C^∊^ atoms). Representative Phe32 and Lys37 atoms were selected to illustrate these trends.

## 4. Discussion   

Here, MX radiation-induced specific structural changes within the large TRAP–RNA assembly over a large dose range (1.3–25.0 MGy) have been analysed using a high-throughput quantitative approach, providing a measure of the electron-density distribution for each refined atom with increasing dose, *D*_loss_. Compared with previous studies, the results provide a further step in the detailed characterization of SRD effects in MX. Our method­ology, which eliminated tedious and error-prone visual inspection, permitted the determination on a per-atom basis of the most damaged sites, as characterized by *F*_obs_(*d*_*n*_) − *F*_obs_(*d*_1_) Fourier difference map peaks between successive data sets collected from the same crystal. Here, it provided the precision required to quantify the role of RNA in the damage susceptibilities of equivalent atoms between RNA-bound and nonbound TRAP, but it is applicable to any MX SRD study.

The RNA was found to be substantially more radiation-resistant than the protein, even at the highest doses investigated (∼25.0 MGy), which is in strong concurrence with our previous SRD investigation of the C.Esp1396I protein–DNA complex (Bury *et al.*, 2015 ▸). Consistent with that study, at high doses of above ∼20 MGy, *F*_obs_(*d*_*n*_) − *F*_obs_(*d*_1_) map density was detected around P, O3′ and O5′ atoms of the RNA backbone, with no significant difference density localized to RNA ribose and basic subunits. RNA backbone disordering thus appears to be the main radiation-induced effect in RNA, with the protein–base interactions maintained even at high doses (>20 MGy). The U4 phosphate exhibited marginally larger *D*_loss_ values above 20 MGy than G1, A2 and G3 (Supplementary Fig. S4). Since U4 is the only refined nucleotide not to exhibit significant base–protein interactions around TRAP (with a water-mediated hydrogen bond detected in only three of the 11 subunits and a single Arg58 hydrogen bond suggested in a further four subunits), this increased U4 *D*_loss_ can be explained owing to its greater flexibility. At 25.0 MGy, the magnitude of the RNA backbone *D*_loss_ was of the same order as for the radiation-insensitive Gly C^α^ atoms and on average less than half that of the acidic residues of the protein (Supplementary Fig. S3). Consequently, no clear single-strand breaks could be located, and since RNA-binding within the current TRAP–(GAGUU)_10_GAG complex is mediated predominantly through base–protein interactions, the biological integrity of the RNA complex was dictated by the rate at which protein decarboxylation occurred.

RNA interacting with TRAP was shown to offer significant protection against radiation-induced structural changes. Both Glu36 and Asp39 bind directly to RNA, each through two hydrogen bonds to guanine bases (G3 and G1, respectively). However, compared with Asp39, Glu36 is strikingly less decarboxylated when bound to RNA (Fig. 4 ▸). This is in good agreement with previous mutagenesis and nucleoside analogue studies (Elliott *et al.*, 2001 ▸), which indicated that the G1 nucleotide does not bind to TRAP as strongly as do A2 and G3, and plays little role in the high RNA-binding affinity of TRAP (*K*_d_ ≃ 1.1 ± 0.4 n*M*). For Glu36 and Asp39, no direct quantitative correlation could be established between hydrogen-bond length and *D*_loss_ (linear *R* ^2^ of <0.23 for all doses; Supplementary Fig. S5). Thus, another factor must be responsible for this clear reduction in Glu36 CO_2_ decarboxyl­ation in RNA-bound TRAP. The Glu36 carboxyl side chain also potentially forms hydrogen bonds to His34 and Lys56, but since these interactions are conserved irrespective of G3 nucleotide binding, this cannot directly account for the stabilization effect on Glu36 in RNA-bound TRAP. Radiation-induced decarboxylation has been proposed to be mediated by preferential positive-hole migration to the side-chain carboxyl group, with rapid proton transfer trapping the hole at the carboxyl group (Burmeister, 2000 ▸; Symons, 1997 ▸):
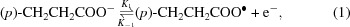
where the forward rate is *K*_1_ and the backward rate is *K*_−1_, 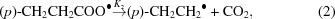
where the forward rate is *K*_2_.

When bound to RNA, the average solvent-accessible area of the Glu36 side-chain O atoms is reduced from ∼15 to 0 Å^2^. We propose that with no solvent accessibility Glu36 decarboxylation is inhibited, since the CO_2_-formation rate *K*_2_ is greatly reduced, and suggest that steric hindrance prevents each radicalized Glu36 CO_2_ group from achieving the planar conformation required for complete dissociation from TRAP. The electron-recombination rate *K*_−1_ remains high, however, owing to rapid electron migration through the protein–RNA complex to refill the Glu36 positive hole (the precursor for Glu decarboxylation). Upon RNA binding, the Asp39 side-chain carboxyl group solvent-accessible area changes from ∼75 to 35 Å^2^, still allowing a high CO_2_-formation rate *K*_2_.

Previous studies have reported inconsistent results concerning the dependence of the acidic residue decarboxylation rate on solvent accessibility (Weik *et al.*, 2000 ▸; Fioravanti *et al.*, 2007 ▸; Gerstel *et al.*, 2015 ▸). The prevalence of radical attack from solvent channels surrounding the protein in the crystal is a questionable cause, considering previous observations indicating that the strongly oxidizing hydroxyl radical is immobile at 100 K (Allan *et al.*, 2013 ▸; Owen *et al.*, 2012 ▸). Furthermore, the suggested electron hole-trapping mechanism which induces decarboxylation within proteins at 100 K has no clear mechanistic dependence on the solvent-accessible area of each carboxyl group. By comparing equivalent acidic residues with and without RNA, we have now deconvoluted the role of solvent accessibility from other local protein environment factors, and thus propose a suitable mechanism by which exceptionally low solvent accessibility can reduce the rate of decarboxylation. Overall, no direct correlation between solvent accessibility and decarboxylation susceptibility was observed, but it is very clear that inaccessible residues are protected.

Apart from these RNA-binding interfaces, RNA binding was seen to enhance decarboxylation for residues Glu50, Glu71 and Glu73, all of which are involved in crystal contacts between TRAP rings (Fig. 4 ▸ *c*). However, for each of these residues the exact crystal contacts are not preserved between bound and nonbound TRAP or even between monomers within one TRAP ring. For example, in bound TRAP, Glu73 hydrogen-bonds to a nearby lysine on each of the 11 subunits, whereas in nonbound TRAP no such interaction exists and Glu73 interacts with a variable number of refined waters in each subunit. Thus, the dependence of decarboxylation rates on these interactions could not be established.

Radiation-induced side-chain conformational changes have been poorly characterized in previous SRD investigations owing to their strong dependence on packing density and geometric strain. Such structural changes are known to have significant roles within enzymatic pathways, and experimenters must be aware of these possible confounding factors when assigning true functional mechanisms using MX. Our results show that RNA binding to TRAP physically stabilizes non-acidic residues within the TRAP macromolecule, most notably Lys37 and Phe32, which stack against the G1 and G3 bases, respectively. It has been suggested (Burmeister, 2000 ▸) that Tyr residues can lose their aromatic –OH group owing to radiation-induced effects; however, no energetically favourable pathway for –OH cleavage exists and this has not been detected in aqueous radiation-chemistry studies. In TRAP, *D*_loss_ increased at a similar rate for both the Tyr O atoms and aromatic ring atoms, suggesting that full ring conformational disordering is more likely. Indeed, no convincing reproducible Fourier difference peaks above the background map noise were observed around any Tyr terminal –OH groups.

The RNA-stabilization effects on protein are observed at short ranges and are restricted to within the RNA-binding interfaces around the TRAP ring. For example, Asp17 is located ∼6.8 Å from the G1 base, outside the RNA-binding interfaces, and has indistinguishable C^γ^ atom *D*_loss_ dose-dynamics between RNA-bound and nonbound TRAP (*p* > 0.9). An increase in the dose at which functionally important residues remain intact has biological ramifications for understanding the mechanisms at which ionizing radiation damage is mitigated within naturally forming DNA–protein and RNA–protein complexes. Observations of lower protein radiation-sensitivity in DNA-bound forms have been recorded in solution at RT at much lower doses (∼1 kGy) than those used for typical MX experiments [*e.g.* an oestrogen response element–receptor complex (Stísová *et al.*, 2006 ▸) and a DNA glycosylase and its abasic DNA target site (Gillard *et al.*, 2004 ▸)]. In these studies, the main damaging species is predicted to be the oxidizing hydroxyl radical produced through solvent irradiation, which is known to add to double covalent bonds within both DNA and RNA bases to induce strand breaks and base modification (Spotheim-Maurizot & Davídková, 2011 ▸; Chance *et al.*, 1997 ▸). It was suggested that physical screening of DNA by protein shielded the DNA–protein interaction sites from radical damage, yielding an extended life-dose for the nucleoprotein complex compared with separate protein and DNA constituents at RT.

However, in the current MX study at 100 K, the main damaging species are believed to be migrating LEEs and holes produced directly within the protein–RNA components or in closely associated solvent. The results presented here suggest that biologically relevant nucleoprotein complexes also exhibit prolonged life-doses under the effect of LEE-induced structural changes, involving direct physical protection of key RNA-binding residues. Such reduced radiation-sensitivity in this case ensures that the interacting protein remains bound long enough to the RNA to complete its function, even whilst exposed to ionizing radiation. Within the nonbound TRAP macromolecule, the acidic residues within the unoccupied RNA-binding interfaces (Asp39, Glu36, Glu42) are notably amongst the most susceptible residues within the asymmetric unit (Fig. 4 ▸). When exposed to X-rays, these residues will be preferentially damaged by X-rays and subsequently reduce the affinity with which TRAP binds to RNA. Within the cellular environment, this mechanism could reduce the risk that radiation-damaged proteins might bind to RNA, thus avoiding the detrimental introduction of incorrect DNA-repair, transcriptional and base-modification pathways.

The Python scripts written to calculate the per atom *D*_loss_ metric are available from the authors on request.

## 5. Related literature   

The following references are cited in the Supporting Information for this article: Chen *et al.* (2010 ▸).

## Supplementary Material

PDB reference: TRAP–RNA, dose 1.3 MGy, 5eeu

PDB reference: dose 3.9 MGy, 5eev

PDB reference: dose 6.5 MGy, 5eew

PDB reference: dose 9.0 MGy, 5eex

PDB reference: dose 11.6 MGy, 5eey

PDB reference: dose 14.1 MGy, 5eez

PDB reference: dose 16.7 MGy, 5ef0

PDB reference: dose 19.3 MGy, 5ef1

PDB reference: dose 25.0 MGy, 5ef3

PDB reference: dose 21.9 MGy, 5ef2

Supporting Information.. DOI: 10.1107/S2059798316003351/rr5121sup1.pdf
