# Supplementary material for: RNA protects a nucleoprotein complex against radiation damage
Source: Acta Crystallogr D Struct Biol. 2016 Apr 26;72(Pt 5):648–57. doi: 10.1107/S2059798316003351 (PMC4854314; doi:10.1107/S2059798316003351)
Supplement: Supplementary file 1 [file d-72-00648-sup1.pdf]

STRUCTURAL BIOLOGY

Volume 72 (2016)

Supporting information for article:

RNA protects a nucleoprotein complex against radiation damage

Charles S. Bury, John E. McGeehan, Alfred A. Antson, Ian Carmichael, Markus Gerstel, Mikhail B. Shevtsov and Elspeth F. Garman

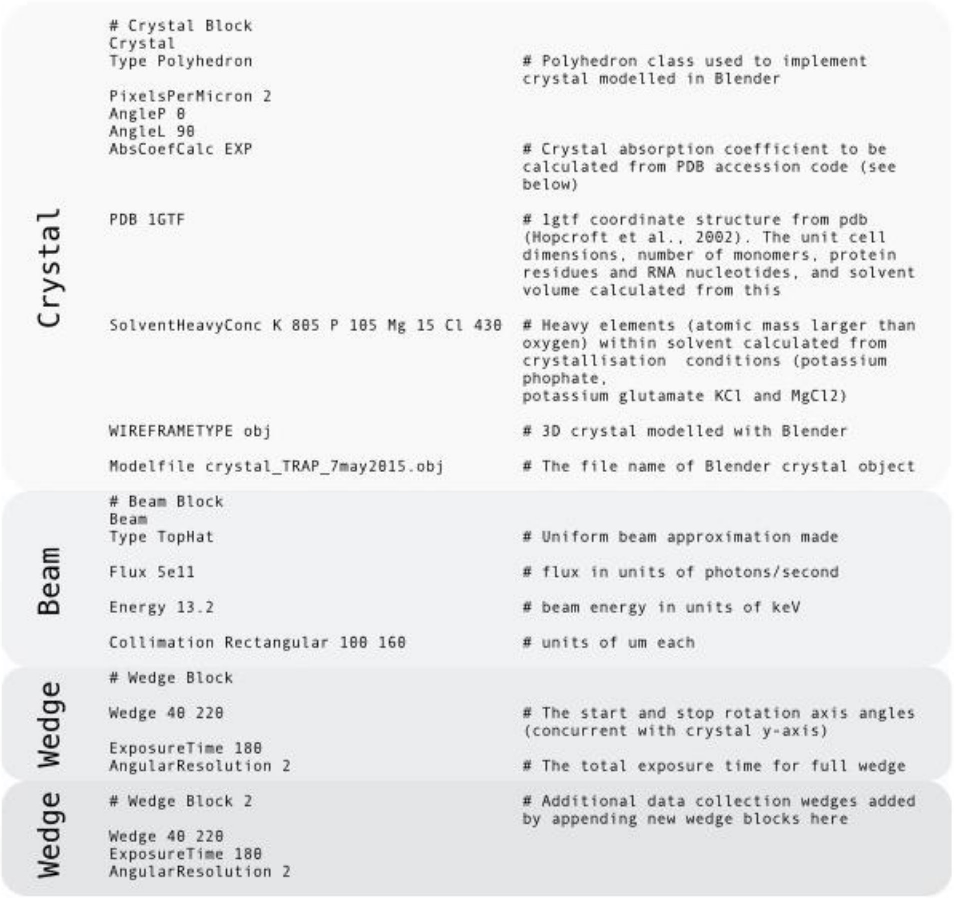

```
Crystal
# Crystal Block
Crystal
Type Polyhedron                              # Polyhedron class used to implement
                                             crystal modelled in Blender
PixelsPerMicron 2
AngleP 0
AngleL 90
AbsCoefCalc EXP                              # Crystal absorption coefficient to be
                                             calculated from PDB accession code (see
                                             below)
PDB 1GTF                                     # 1gtf coordinate structure from pdb
                                             (Hopcroft et al., 2002). The unit cell
                                             dimensions, number of monomers, protein
                                             residues and RNA nucleotides, and solvent
                                             volume calculated from this
SolventHeavyConc K 805 P 105 Mg 15 Cl 430    # Heavy elements (atomic mass larger than
                                             oxygen) within solvent calculated from
                                             crystallisation  conditions (potassium
                                             phophate,
                                             potassium glutamate KCl and MgCl2)
WIREFRAMETYPE obj                            # 3D crystal modelled with Blender
Modelfile crystal_TRAP_7may2015.obj          # The file name of Blender crystal object

Beam
# Beam Block
Beam
Type TopHat                                  # Uniform beam approximation made
Flux 5e11                                    # flux in units of photons/second
Energy 13.2                                  # beam energy in units of keV
Collimation Rectangular 100 160              # units of um each

Wedge
# Wedge Block
Wedge 40 220                                 # The start and stop rotation axis angles
                                             (concurrent with crystal y-axis)
ExposureTime 180
AngularResolution 2                          # The total exposure time for full wedge

Wedge
# Wedge Block 2                              # Additional data collection wedges added
                                             by appending new wedge blocks here
Wedge 40 220
ExposureTime 180
AngularResolution 2
```

Figure S1 RADDOSE-3D (Zeldin, Gerstel, *et al.*, 2013) .txt input file for TRAP dataset 2, calculating a DWD (Diffraction-Weighted Dose (Zeldin, Brockhauser, *et al.*, 2013)) of 3.88 MGy. Crystal, beam and data collection wedge parameters are defined in three discrete input blocks. Additional data collection wedges are described by appending adding additional wedge blocks to the input file (here 2 successive 100 degree wedge of 1° images over the same angular range are simulated).

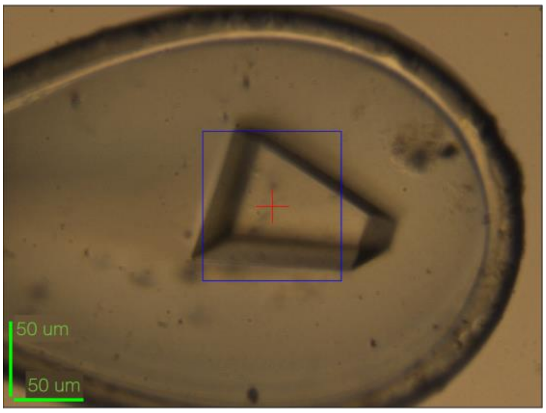

(a)

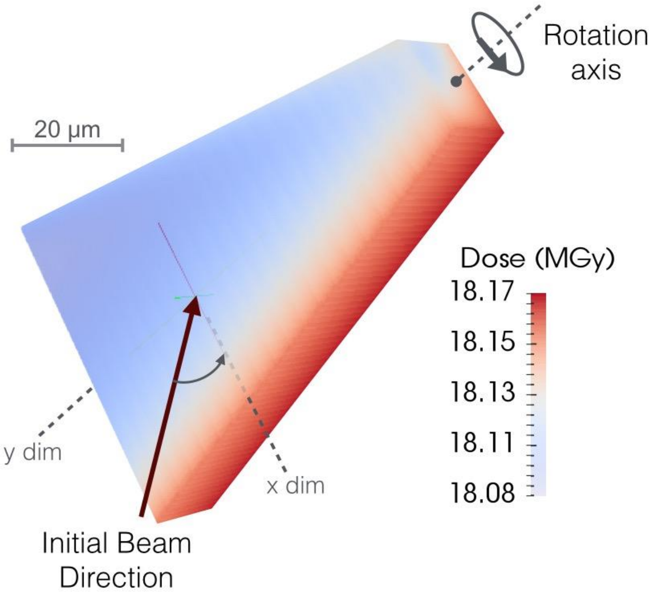

(b)

Figure S2 (a) The TRAP-RNA crystal within a rayon cryo-loop on beamline. The *x* and *y* crystal dimensions were estimated at 60 µm, 80 µm, 80 µm, 30 µm (travelling in a clockwise direction around the crystal from the left-most side). The z dimension (~µm) is orientated directly into the page, perpendicular to the loop. The red crosshair indicates the beam centre, and the blue reference box shown around the crystal is 100 µm × 100 µm. (b) The dose distribution within the TRAP crystal calculated in RADDOSE-3D for the 7th dataset (DWD: 16.7 MGy). The initial beam direction and rotation axis (concurrent with y-axis) are shown. At each dataset a homogenous dose distribution (to within ~ 0.51%) was predicted throughout the crystal.

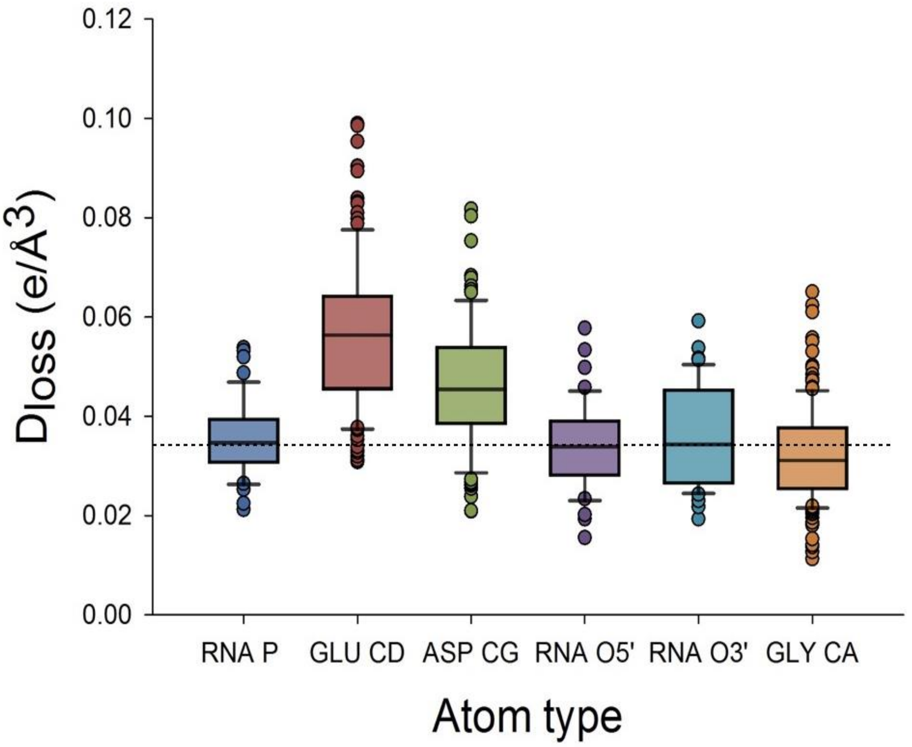
0.12
0.10
0.08
0.06
0.04
0.02
0.00
Dloss (e/Å3)
RNA P
GLU CD
ASP CG
RNA O5'
RNA O3'
GLY CA
Atom type

(a)

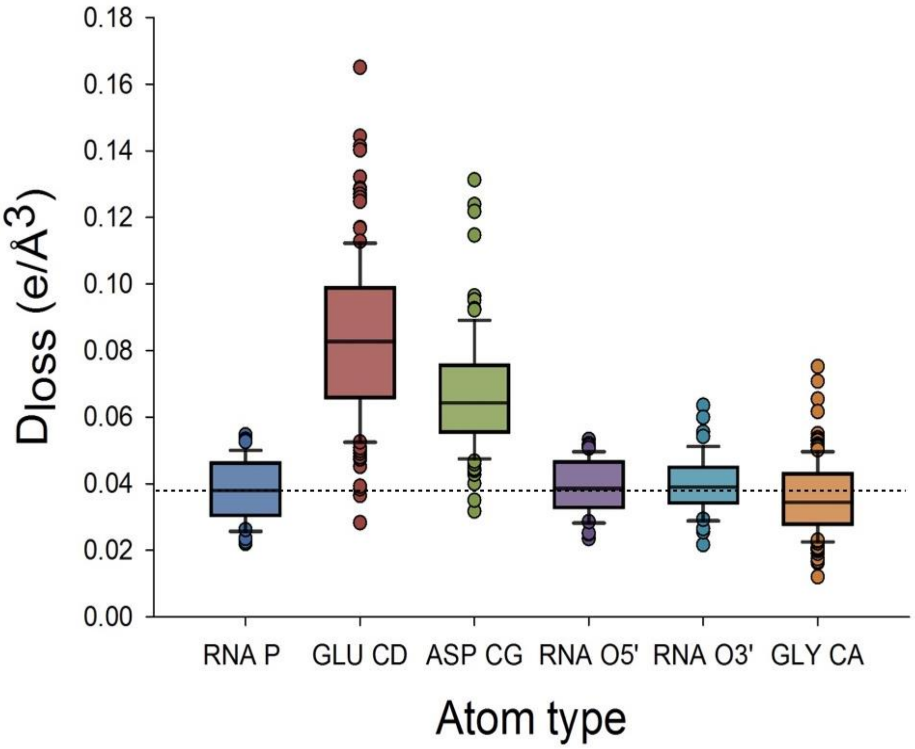
0.18
0.16
0.14
0.12
0.10
0.08
0.06
0.04
0.02
0.00
Dloss (e/Å3)
RNA P
GLU CD
ASP CG
RNA O5'
RNA O3'
GLY CA
Atom type

(b)

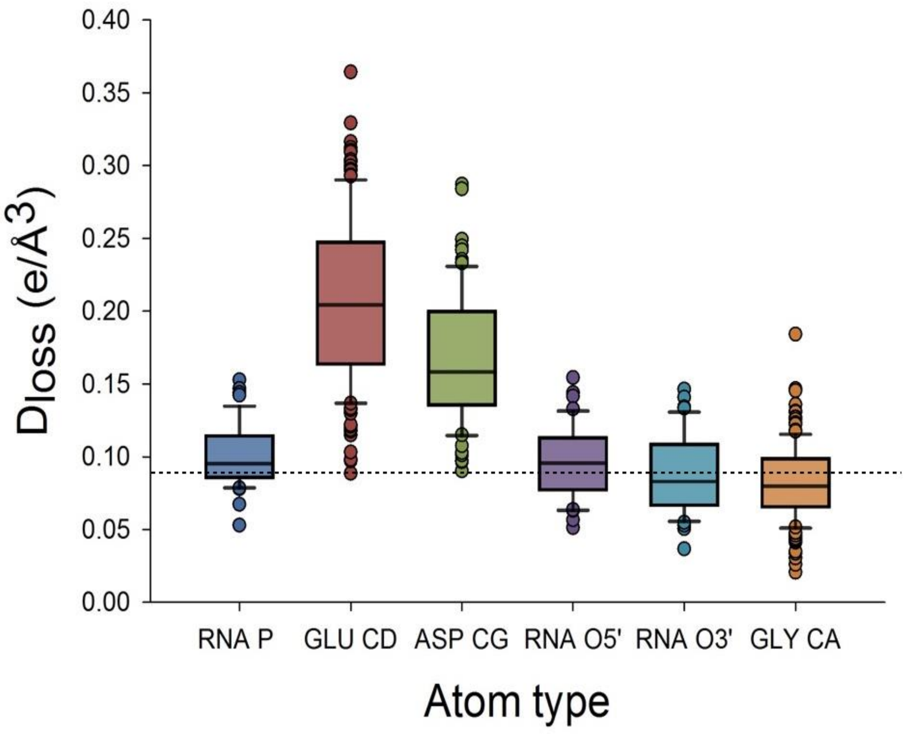

(c)

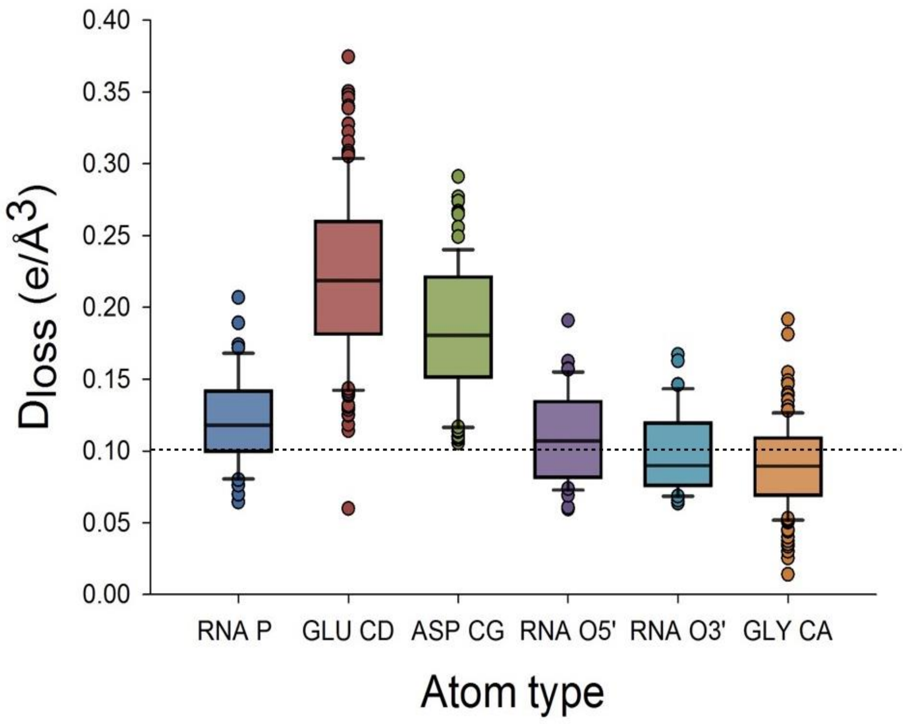

(d)

Figure S3 D_{loss} metric calculated for all RNA P, O5' and O3' atoms, and all Glu C_δ, Asp C_γ and Gly C_α atoms over 4 increasing doses (a) 3.88 MGy, (b) 6.45 MGy, (c) 21.9 MGy and (d) 25.0 MGy. The Gly C_α atom control is included as a measure of the overall D_{loss} increase with dose due to global radiation damage effects. The horizontal dashed line illustrates the average D_{loss} calculated over all

refined atoms in the TRAP complex, at each dose, as a measure of the overall rate of density disordering due to *global radiation damage*. Over the large dose range, RNA backbone atoms were determined to disorder at a rate on the same order as the overall global damage, in contrast with Glu and Asp residues, which consistently disordered at a rate above the global damage rate.

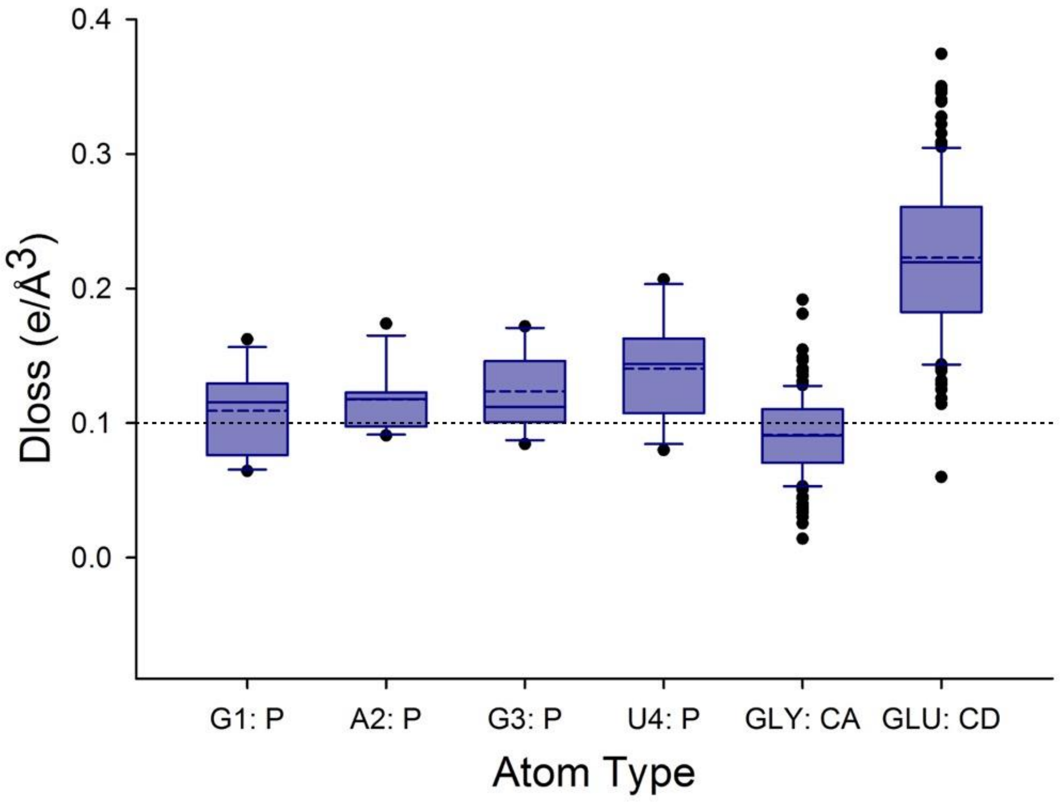

Figure S4 D_{loss} metric calculated for all phosphorus atoms for each of 4 refined nucleotide types: G1, A2, G3 and U4 at the highest investigated dose (25.0 MGy). The D_{loss} calculated for Glu C_{δ} and Gly C_{α} atoms is included for comparison. The U4 nucleotide does not interact directly with the protein, and the backbone phosphorus atom of U4 was shown to disorder on average at a rate marginally lower than that of the other refined nucleotide types (G1, A2, G3). However the U4 P atom disordering is still significantly lower than that of Glu side-chain carboxyl atoms. The horizontal dashed line illustrates the average D_{loss} calculated over all refined atoms in the TRAP complex, as a measure of the overall rate of density disordering due to *global radiation damage* effects.

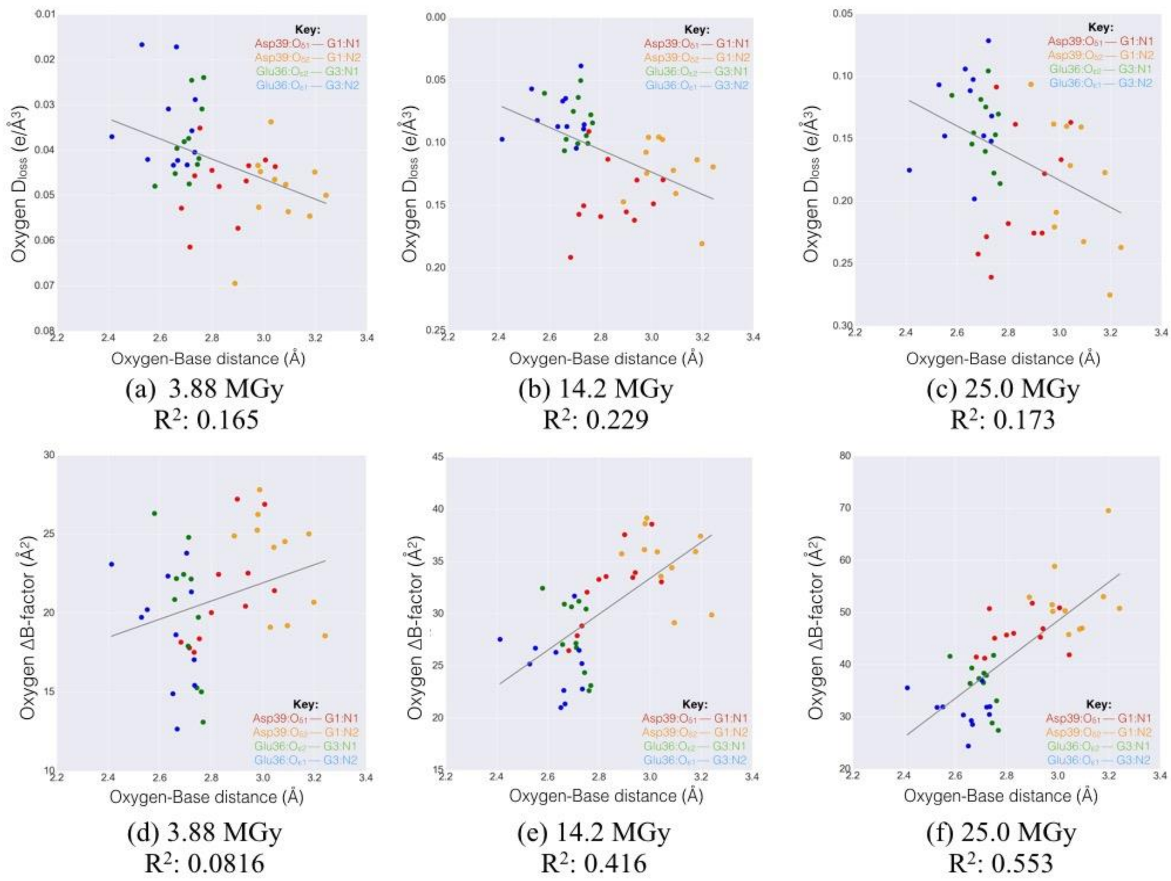

Figure S5 (a-c) D_{loss} and (d-f) ΔB-factor (change in atomic B-factor between dataset n and 1) metrics calculated for Asp 39 and Glu 36 side-chain carboxyl oxygen atoms, plotted against hydrogen bonding distance (Å) to the nearest G1 and G3 base respectively. Plots are given for 3 increasing doses between 3.88 and 25.0 MGy. Linear regression R^2 values for each scatter plot are given. With increasing dose, the ΔB-factor metric was observed to become increasingly linearly correlated with carboxyl oxygen-base hydrogen bonding length; the D_{loss} metric however did not correlate with hydrogen bond length. The Asp-39 side-chain carboxyl oxygens have been observed in previous studies to consistently make one or two distorted hydrogen bonds with the N1 and N2 of the G1 RNA base around the TRAP ring, depending on the exact RNA binding sequence (Hopcroft *et al.*, 2002, Antson *et al.*, 1999, Elliott *et al.*, 2001). For the Asp-39-G1 base interactions observed here, the mean $O_{\delta1}$–N1 interaction distance is 2.9 Å whereas the $O_{\delta2}$–N2 is consistently larger, with a mean distance of 3.1 Å. For Asp-39, only for the Oδ1 carboxyl oxygen were the D_{loss} dynamics statistically distinguishable between bound and non-RNA TRAP (Hotelling T-Squared Test: $O_{\delta1}$ p=0.019 compared to $O_{\delta2}$ p=0.109). These observations are in agreement with those made previously, in which Asp-39 was reported to interact with the G1 base only through a single distorted $O_{\delta1}$–N2 hydrogen bond (Hopcroft *et al.*, 2002). The Glu-36-G3 base hydrogen bond interactions were notably shorter, with mean interaction distances of 2.6 Å ($O_{\varepsilon1}$–N2) and 2.7 Å ($O_{\varepsilon2}$–N1). An increasing correlation with dose was observed between oxygen-base hydrogen bond length and ΔB-factor ($R^2 > 0.41$ for dose

≥11.6 MGy) for the individual Glu-36 and Asp-39 residues. However no direct quantitative correlation could be established between hydrogen bond length and D_{loss} (linear $R^2 < 0.23$ for all doses).

Table S1 Data processing and refinement statistics. Values in parentheseses are for the highest-resolution shells. For observed F_{obs} and calculated F_{calc} structure factors, $R_{work} = \sum|F_{obs} - F_{calc}| / \sum F_{obs}$, and R_{free} is the R_{work} formula calculated from the same 5% test set of randomly selected reflections excluded throughout refinement. The current TRAP-RNA structure crystallised in space group C2 (α = γ = 90°). The resolution range was fixed at 63.00 - 1.98 Å throughout, with 2.01 - 1.98 Å for the outer shell for all datasets. *MolProbity* (Chen *et al.*, 2010) was used for structure validation within *phenix.refine*, giving for all structures: RMSD bond length: 0.024 Å, RMSD bond angle: 2.3°, Ramachandran favoured/outliers/allowed: 99.5/0.0/0.5%, rotamer outliers: 2.6%. The number of protein, RNA and solvent non-hydrogen atoms remained constant at 12135, 968 and 743 respectively for all dataset numbers, due to the rigid body refinement of higher dose datasets.

Dataset	1	2	3	4	5	6	7	8	9	10
DWD (MGy)	1.31	3.88	6.45	9.02	11.58	14.15	16.72	19.29	21.86	24.98
Data processing										
Cell dimensions										
a (Å)	140.90	141.00	141.04	141.04	141.12	141.14	141.16	141.19	141.21	141.23
b (Å)	110.89	110.98	111.02	111.05	111.08	111.10	111.13	111.17	111.16	111.16
c (Å)	137.81	137.93	137.99	138.05	138.09	138.15	138.18	138.21	138.28	138.29
β (°)	117.41	117.41	117.41	117.40	117.40	117.39	117.39	117.39	117.39	117.37
# observs.	473080	471462	472091	472756	473225	476499	477213	477019	475519	472868

Dataset	1	2	3	4	5	6	7	8	9	10
# Unique reflc.	129942	130202	130317	130454	130599	130738	130870	130920	130982	130840
Multiplicity	3.6 (3.5)	3.6 (3.4)	3.6 (3.4)	3.6 (3.4)	3.6 (3.4)	3.6 (3.4)	3.6 (3.4)	3.6 (3.4)	3.6 (3.4)	3.6 (3.4)
Completeness (%)	99.3	99.3	99.3	99.3	99.3	99.3	99.3	99.3	99.3	99.1
R_{merge}	0.08 (0.70)	0.08 (0.78)	0.08 (0.86)	0.09 (1.02)	0.09 (1.13)	0.10 (1.31)	0.10 (1.50)	0.11 (1.68)	0.11 (2.07)	0.11 (1.98)
Mean((I)/sd(I))	9.6 (1.5)	9.4 (1.4)	9.3 (1.2)	8.8 (1.1)	8.4 (1.0)	8.1 (0.8)	7.4 (0.7)	7 (0.6)	7 (0.5)	7.2 (0.5)
I_n/I_1	1	0.905	0.865	0.831	0.776	0.738	0.676	0.638	0.625	0.854
Refinement										
R_{work}	0.192	0.209	0.209	0.212	0.216	0.220	0.225	0.230	0.234	0.240
R_{free}	0.231	0.244	0.243	0.245	0.248	0.252	0.256	0.263	0.264	0.271
Mean B-factor (Å^2)	33.78	34.50	36.04	37.22	38.86	40.35	42.89	44.70	47.71	47.58

Antson, A. A., Dodson, E. J., Dodson, G., Greaves, R. B., Chen, X. & Gollnick, P. (1999). *Nature* **401**, 235-242.

Chen, V. B., Arendall, W. B., Headd, J. J., Keedy, D. A., Immormino, R. M., Kapral, G. J., Murray, L. W., Richardson, J. S. & Richardson, D. C. (2010). *Acta Cryst.* D**66**, 12-21.

Elliott, M. B., Gottlieb, P. A. & Gollnick, P. (2001). *RNA* **7**, 85-93.

Hopcroft, N. H., Wendt, A. L., Gollnick, P. & Antson, A. A. (2002). *Acta Cryst.* D**58**, 615-621.

Zeldin, O. B., Brockhauser, S., Bremridge, J., Holton, J. M. & Garman, E. F. (2013). *Proc. Natl Acad. Sci. USA* **110**, 20551-20556.

Zeldin, O. B., Gerstel, M. & Garman, E. F. (2013). *J. Appl. Cryst.* **46**, 1225-1230.
